# Supplementary material for: Prevalence of questionable research practices, research misconduct and their potential explanatory factors: A survey among academic researchers in The Netherlands
Source: PLoS One. 2022 Feb 16;17(2):e0263023. doi: 10.1371/journal.pone.0263023 (PMC8849616; doi:10.1371/journal.pone.0263023)
Supplement: S1 Fig — a. Flowchart of supporting institutions (n = 8). b. Flowchart of non-supporting institutions (n = 14). (DOCX) [file pone.0263023.s001.docx]

# S1a Fig. Flowchart of supporting institutions (n = 8)

Total number Invited from supporting institutions

N = 23,094

N_adj_ = 20,879

Bounced

N = 22

No response

N= 17,925

Opted out

N = 231

Completed the survey

N= 3270

Opened survey link

N= 4904

Ineligible

N = 466

Did not complete the survey

N= 1144

Did not consent

N = 24

N= 78

Eligible responders who started the survey

N= 4414

**3258 1151 = 4409**

N_adj_ = total number of email addresses provided to us by the supporting institutions with three corrections:

a) Inclusion of a number of eligible researchers who were not included in the original e-mail list provided to Kantar by their institutions

b) deducting the no. of bounced e-mail addresses and

c) applying a correction factor based on the number of participations who did not fit our inclusion criteria

We defined c) as the fraction of invitees who opened the survey link and consented to participate but subsequently turned out to be ineligible i.e. did not meet the inclusion criteria (Fig 1):

466 / 4904 = 0.095

This calculation is based on the assumption that eligibility does not influence the decision to open the survey and to provide informed consent. That seems to be a reasonable assumption because the exact eligibility criteria were obscure for invitees until they had opened the survey and provided informed consent. Therefore, taken together, our best estimate of the total number of eligible invitees that fit our inclusion criteria from supporting institutes can be calculated as follows:

(23094 – 22) – (0.095 * 23094) = 20879 (N_adj_)

% Response = 4414/20879 = 21.1%

# S1b Fig. Flowchart of non-supporting institutions (n = 14)

Total number Invited from non-supporting institutions

N= 40,684

Bounced

N= 1891

No response

N= 32,099

Opted out

N= 710

Completed surveys

N= 3543

Opened survey link

N= 5957

Ineligible

N= 788

Did not complete the survey

N= 1563

Did not consent

N=54

Eligible responders who started the survey

N = 5106
