# Supplementary figures and images for: Prevalence of questionable research practices, research misconduct and their potential explanatory factors: A survey among academic researchers in The Netherlands
Source: PLoS One. 2022 Feb 16;17(2):e0263023. doi: 10.1371/journal.pone.0263023 (PMC8849616; doi:10.1371/journal.pone.0263023)

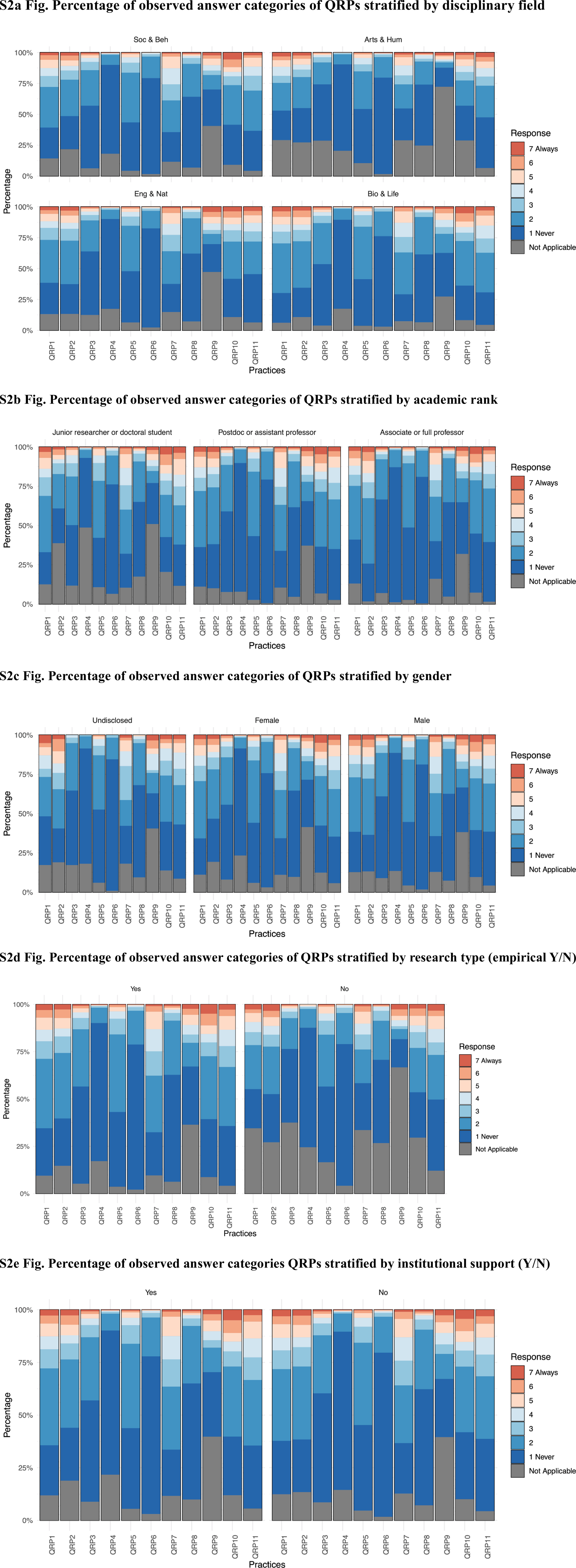

Supplement: S2 Fig — a. Percentage of observed answer categories of QRPs stratified by disciplinary field. b. Percentage of observed answer categories of QRPs stratified by academic rank. c. Percentage of observed answer categories of QRPs stratified by gender. d. Percentage of observed answer categories of QRPs stratified by research type (empirical Y/N). e. Percentage of observed answer categories QRPs stratified by institutional support (Y/N). (TIF) [file pone.0263023.s002.tif]

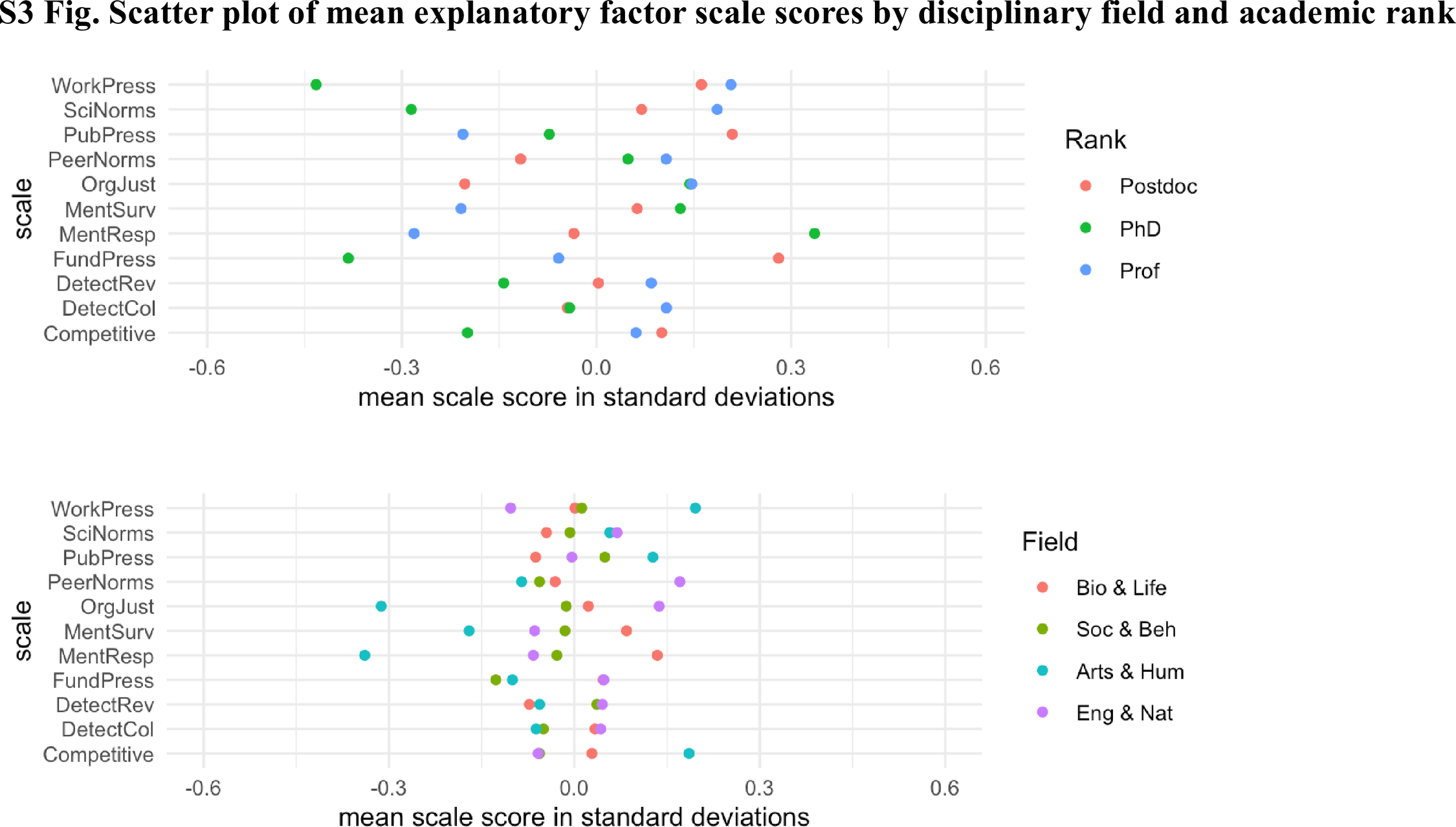

Supplement: S3 Fig — (TIF) [file pone.0263023.s003.tif]
