# Supplementary material for: Prevalence of questionable research practices, research misconduct and their potential explanatory factors: A survey among academic researchers in The Netherlands
Source: PLoS One. 2022 Feb 16;17(2):e0263023. doi: 10.1371/journal.pone.0263023 (PMC8849616; doi:10.1371/journal.pone.0263023)
Supplement: S1 Table — (DOCX) [file pone.0263023.s004.docx]

# S1 Table. Characteristics of all respondents by disciplinary field, academic rank, gender, research type and institutional support

|  | **Disciplinary field** | | | | **Academic rank** | | | **Total** |
| --- | --- | --- | --- | --- | --- | --- | --- | --- |
|  | **Life and**  **medical**  **sciences**  **(N = 2747)** | **Social and behavourial**  **sciences**  **(N = 1965)** | **Natural and**  **engineering**  **sciences**  **(N = 1465)** | **Arts and**  **humanities**  **(N = 636)** | **PhD candidates and junior researchers**  **(N = 2013)** | **Postdocs and assistant professors**  **(N = 2733)** | **Associate and full professors**  **(N = 2066)** | **Total sample**  **(N = 6813)** |
| **Female (%)** | 48.7 | 51.5 | 24.9 | 46.1 | 56.9 | 46.3 | 28.8 | 44.2 |
| **Male (%)** | 49.6 | 47.0 | 73.5 | 50.8 | 41.9 | 51.7 | 69.3 | 54.1 |
| **Undisclosed (%)** | 1.6 | 1.5 | 1.6 | 3.1 | 1.2 | 1.9 | 1.9 | 1.7 |
| **Being mainly engaged in empirical research (%)** | 97.6 | 94.0 | 77.7 | 65.1 | 87.9 | 89.6 | 90.2 | 89.3 |
| **Institutional support (%)** | 58.6 | 52.3 | 23.5 | 45.3 | 59.6 | 45.2 | 40.5 | 48.0 |
