# Supplementary material for: Prevalence of questionable research practices, research misconduct and their potential explanatory factors: A survey among academic researchers in The Netherlands
Source: PLoS One. 2022 Feb 16;17(2):e0263023. doi: 10.1371/journal.pone.0263023 (PMC8849616; doi:10.1371/journal.pone.0263023)
Supplement: S2 Table — (DOCX) [file pone.0263023.s005.docx]

# S2 Table. Prevalence (%) of the “not applicable” answers stratified by disciplinary field and academic rank

|  | |  | |  | **Disciplinary field** | | |  | | **Academic rank** | | |
| --- | --- | --- | --- | --- | --- | --- | --- | --- | --- | --- | --- | --- |
| **QRP** | **Description (In the last three years..)** | | **Life and medical sciences** | | **Social and behavioural sciences** | **Natural and engineering sciences** | **Arts and humanities** | | **PhD candidates and junior researchers** | | **Postdocs and**  **assistant professors** | **Associate and full professors** |
| **QRP1** | Insufficient attention to the equipment, skills or expertise | | 6.2 | | 14.2 | 13.1 | 29.1 | | 12.6 | | 11.1 | 13.1 |
| **QRP2** | Insufficiently supervised or mentored junior co-workers | | 10.8 | | 21.7 | 13.3 | 27.2 | | 38.7 | | 10.1 | 1.7 |
| **QRP3** | Inadequate research designs or unsuitable measurement instruments | | 3.9 | | 6.2 | 12.5 | 28.6 | | 11.8 | | 7.7 | 7.0 |
| **QRP4** | Unfairly reviewed manuscripts, grant applications or colleagues | | 17.5 | | 18.1 | 17.4 | 20.4 | | 48.7 | | 7.9 | 1.2 |
| **QRP5** | Conclusions not sufficiently substantiated | | 3.7 | | 4.2 | 6.3 | 10.4 | | 10.7 | | 2.6 | 2.7 |
| **QRP6** | Improper referencing of source | | 3.0 | | 1.6 | 2.3 | 1.6 | | 6.5 | | 0.6 | 0.5 |
| **QRP7** | Inadequate notes of research process | | 7.4 | | 11.5 | 14.8 | 28.9 | | 10.4 | | 10.5 | 16.1 |
| **QRP8** | Failed to report important study details in publications | | 6.6 | | 6.8 | 7.2 | 24.7 | | 17.4 | | 4.6 | 4.8 |
| **QRP9** | Not submitting or resubmit valid negative studies for publication | | 27.4 | | 40.6 | 47.3 | 72.3 | | 50.9 | | 37.2 | 31.9 |
| **QRP10** | Insufficient inclusion of study flaws and limitations in publications | | 8.3 | | 9.1 | 10.8 | 28.8 | | 20.4 | | 6.8 | 7.3 |
| **QRP11** | Selectively cited references to enhance findings or convictions | | 4.4 | | 4.2 | 6.3 | 6.4 | | 11.6 | | 2.6 | 1.5 |
