# Supplementary material for: Prevalence of questionable research practices, research misconduct and their potential explanatory factors: A survey among academic researchers in The Netherlands
Source: PLoS One. 2022 Feb 16;17(2):e0263023. doi: 10.1371/journal.pone.0263023 (PMC8849616; doi:10.1371/journal.pone.0263023)
Supplement: S3 Table. a. Mean score (95% confidence interval) of QRPs stratified by disciplinary field and academic rank. b. Mean score (95% confidence interval) and prevalence (95% confidence interval) of QRPs stratified by gender, research type and institutional support — (DOCX) [file pone.0263023.s006.docx]

# S3a Table. Mean score (95% confidence interval) of QRPs stratified by disciplinary field and academic rank

| **QRP** | **Description (In the last three years..)** | **Life and medical sciences**  **(N = 2747; 40.3%)** | **Social and behavioural sciences**  **(N = 1965; 28.8%)** | **Natural and engineering sciences**  **(N = 1465; 21.5%)** | **Arts and humanities**  **(N = 636; 9.3%)** | **PhD candidates and junior researchers**  **(N = 2013; 29.5%)** | **Postdocs and**  **assistant professors**  **(N = 2733; 40.1%)** | **Associate and full professors**  **(N = 2066; 30.3%)** | **Overall**  **(N = 6813; 100%)** |
| --- | --- | --- | --- | --- | --- | --- | --- | --- | --- |
| **QRP1**  **(N = 5987)** | Insufficient attention to the equipment, skills or expertise | 2.5 (2.5,2.6) | 2.5 (2.4,2.6) | 2.4 (2.4,2.5) | 2.6 (2.4,2.7) | 2.6 (2.5,2.7) | 2.5 (2.4,2.6) | 2.4 (2.3,2.5) | 2.5 (2.5,2.5) |
| **QRP2**  **(N = 5722)** | Insufficiently supervised or mentored junior co-workers | 2.5 (2.5,2.6) | 2.4 (2.3,2.4) | 2.5 (2.4,2.6) | 2.3 (2.2,2.4) | 2.3 (2.3,2.4) | 2.4 (2.4,2.5) | 2.6 (2.5,2.7) | 2.5 (2.4,2.5) |
| **QRP3**  **(N = 6221)** | Inadequate research designs or unsuitable measurement instruments | 1.8 (1.7,1.8) | 1.8 (1.7,1.8) | 1.7 (1.6,1.7) | 1.6 (1.5,1.6) | 2.0 (1.9,2) | 1.7 (1.7,1.7) | 1.6 (1.5,1.6) | 1.7 (1.7,1.8) |
| **QRP4**  **(N = 5592)** | Unfairly reviewed manuscripts, grant applications or colleagues | 1.2 (1.1,1.2) | 1.2 (1.1,1.2) | 1.2 (1.1,1.2) | 1.1 (1.1,1.2) | 1.2 (1.2,1.2) | 1.1 (1.1,1.2) | 1.2 (1.2,1.2) | 1.2 (1.2,1.2) |
| **QRP5**  **(N = 6470)** | Conclusions not sufficiently substantiated | 1.9 (1.9,1.9) | 1.9 (1.9,2) | 1.9 (1.8,1.9) | 1.9 (1.8,1.9) | 2.1 (2.0,2.1) | 1.9 (1.8,1.9) | 1.7 (1.7,1.8) | 1.9 (1.9,1.9) |
| **QRP6**  **(N = 6655)** | Improper referencing of source | 1.3 (1.3,1.3) | 1.3 (1.2,1.3) | 1.2 (1.2,1.3) | 1.3 (1.2,1.3) | 1.4 (1.3,1.4) | 1.3 (1.2,1.3) | 1.2 (1.2,1.3) | 1.3 (1.3,1.3) |
| **QRP7**  **(N = 5984)** | Inadequate notes of research process | 2.6 (2.6,2.7) | 2.6 (2.6,2.7) | 2.6 (2.6,2.7) | 2.5 (2.4,2.6) | 2.7 (2.6,2.8) | 2.6 (2.6,2.7) | 2.5 (2.5,2.6) | 2.6 (2.6,2.7) |
| **QRP8**  **(N = 6237)** | Failed to report important study details in publications | 1.6 (1.6,1.6) | 1.6 (1.5,1.6) | 1.6 (1.5,1.6) | 1.6 (1.5,1.7) | 1.6 (1.6,1.7) | 1.6 (1.6,1.6) | 1.5 (1.5,1.6) | 1.6 (1.6,1.6) |
| **QRP9**  **(N = 4111)** | Not submitting or resubmit valid negative studies for publication | 2.3 (2.2,2.3) | 2.3 (2.3,2.4) | 2.8 (2.7,2.9) | 2.4 (2.2,2.5) | 2.3 (2.2,2.4) | 2.5 (2.4,2.6) | 2.3 (2.2,2.4) | 2.4 (2.4,2.4) |
| **QRP10**  **(N = 6067)** | Insufficient inclusion of study flaws and limitations in publications | 2.6 (2.5,2.7) | 2.5 (2.4,2.6) | 2.5 (2.4,2.6) | 2.4 (2.3,2.5) | 2.8 (2.7,2.9) | 2.5 (2.5,2.6) | 2.3 (2.2,2.4) | 2.5 (2.5,2.6) |
| **QRP11**  **(N = 6476)** | Selectively cited references to enhance findings or convictions | 2.6 (2.6,2.7) | 2.4 (2.3,2.4) | 2.3 (2.3,2.4) | 2.3 (2.2,2.4) | 2.7 (2.7,2.8) | 2.5 (2.4,2.5) | 2.2 (2.2,2.3) | 2.5 (2.4,2.5) |

*Scores range from 1=never to 7=always; Mean scores of individual QRPs only consider respondents that deemed the QRP at issue applicable.*

# S3b Table. Mean score (95% confidence interval) and prevalence (95% confidence interval) of QRPs stratified by gender, research type and institutional support

| **QRP** | **Description (In the last three years..)** | **Gender** | | | **Being mainly engaged in empirical research** | **Institutional support** |
| --- | --- | --- | --- | --- | --- | --- |
|  |  | **Male (N = 3689)** | **Female (N = 3008)** | **Undisclosed (N = 116)** | **(N = 6083)** | **(N = 3270)** |
| **QRP1 (N = 5987)** | Insufficient attention to the equipment, skills or expertise essential to perform my studies | 2.5 (2.4,2.5) | 2.5 (2.5,2.6) | 2.5 (2.2,2.8) | 2.5 (2.5,2.6) | 2.5 (2.5,2.6) |
|  |  | 14.6 (13.4,15.9) | 14.8 (13.5,16.2) | 15.6 (9.3,24.8) | 14.8 (13.9,15.7) | 14.3 (13.0,15.6) |
| **QRP2**  **(N = 5722)** | Supervised or mentored junior co-workers | 2.6 (2.5,2.6) | 2.4 (2.3,2.4) | 2.8 (2.5,3.2) | 2.5 (2.4,2.5) | 2.4 (2.4,2.5) |
|  |  | 15.9 (14.7,17.3) | 13.4 (12.1,14.9) | 22.3 (14.7,32.3) | 14.9 (14,15.9) | 14.9 (13.6,16.3) |
| **QRP3**  **(N = 6221)** | Inadequate research designs or used evidently unsuitable measurement instruments for my studies | 1.7 (1.7,1.7) | 1.8 (1.8,1.8) | 1.5 (1.4,1.6) | 1.7 (1.7,1.8) | 1.8 (1.7,1.8) |
|  |  | 4.3 (3.6,5.0) | 4.6 (3.8,5.4) | none | 4.4 (3.9,5) | 4.5 (3.8,5.4) |
| **QRP4**  **(N = 5592)** | Unfairly reviewed manuscripts, grant applications or colleagues applying for promotion. | 1.2 (1.2,1.2) | 1.2 (1.1,1.2) | 1.1 (1.1,1.2) | 1.2 (1.2,1.2) | 1.2 (1.2,1.2) |
|  |  | 0.8 (0.6,1.2) | 0.8 (0.5,1.3) | none | 0.8 (0.6,1.1) | 0.7 (0.4,1.1) |
| **QRP5**  **(N = 6470)** | Drew conclusions that were not sufficiently substantiated by my studies | 1.9 (1.8,1.9) | 1.9 (1.9,1.9) | 1.8 (1.6,1.9) | 1.9 (1.9,1.9) | 1.9 (1.9,1.9) |
|  |  | 4.1 (3.5,4.9) | 3.9 (3.3,4.7) | 2.8 (0.7,8.4) | 3.8 (3.4,4.4) | 4 (3.4,4.8) |
| **QRP6**  **(N = 6655)** | Used published or unpublished ideas or phrases from others without properly referencing its source | 1.2 (1.2,1.3) | 1.3 (1.3,1.4) | 1.2 (1.1,1.3) | 1.3 (1.3,1.3) | 1.3 (1.3,1.3) |
|  |  | 0.6 (0.3,0.9) | 0.8 (0.5,1.2) | none | 0.6 (0.5,0.9) | 0.5 (0.3,0.8) |
| **QRP7**  **(N = 5984)** | Kept inadequate notes of my research process in a project | 2.7 (2.6,2.7) | 2.6 (2.5,2.6) | 2.6 (2.4,2.9) | 2.6 (2.6,2.7) | 2.6 (2.6,2.7) |
|  |  | 15.8 (14.6,17.2) | 13.0 (11.8,14.4) | 12.6 (7.0,21.4) | 14.5 (13.6,15.4) | 14.1 (12.9,15.4) |
| **QRP8**  **(N = 6237)** | Did not mention clearly important details of my study method in my publications | 1.6 (1.6,1.6) | 1.6 (1.6,1.6) | 1.4 (1.3,1.6) | 1.6 (1.6,1.6) | 1.6 (1.5,1.6) |
|  |  | 2.6 (2.1,3.2) | 3.2 (2.6,4.0) | 1.0 (0,6.0) | 2.9 (2.5,3.4) | 2.9 (2.3,3.5) |
| **QRP9**  **(N = 4111)** | Chose not to submit or resubmit valid negative studies for publication | 2.5 (2.5,2.6) | 2.2 (2.2,2.3) | 2.8 (2.5,3.2) | 2.4 (2.3,2.4) | 2.3 (2.3,2.4) |
|  |  | 19.8 (18.2,21.5) | 14.5 (12.9,16.2) | 20.3 (11.9,32) | 17.1 (15.9,18.3) | 16.9 (15.2,18.6) |
| **QRP10**  **(N = 6067)** | Insufficiently mentioned study flaws and limitations in my publications | 2.5 (2.5,2.6) | 2.5 (2.5,2.6) | 2.3 (2.0,2.6) | 2.5 (2.5,2.6) | 2.6 (2.5,2.6) |
|  |  | 17.0 (15.8,18.4) | 17.2 (15.8,18.8) | 10.0 (5.2,18) | 17.1 (16.1,18.1) | 17.1 (15.8,18.5) |
| **QRP11**  **(N = 6476)** | Selectively cited references to enhance my own findings or convictions | 2.4 (2.4,2.5) | 2.5 (2.5,2.6) | 2.4 (2.1,2.7) | 2.5 (2.4,2.5) | 2.5 (2.5,2.6) |
|  |  | 13.8 (12.7,15.0) | 14.4 (13.2,15.8) | 12.3 (7.0,20.4) | 14.0 (13.1,14.9) | 14.4 (13.2,15.7) |
| **Any Frequent QRP** | Score 5, 6 or 7 on at least 1 of the 11 QRPs | 52.7  (51.0, 54.3) | 49.8  (4.8, 51.6) | 48.3  (39.0, 57.7) | 52.5  (51.2, 53.7) | 50.9  (49.2, 52.6) |
| **Falsification** | Making up of data or results | 3.7  (1.8, 5.7) | 5.1  (2.9, 7.2) | 0.9  (0, 11.2) | 4.4  (2.9, 5.9) | 4.4  (2.4, 6.5) |
| **Fabrication** | Manipulating research materials, data or results | 3.7  (1.8, 5.6) | 4.9  (2.8, 7.1) | 3.4  (0, 14.1) | 4.6  (3.1, 6.1) | 3.9  (1.8, 5.9) |
| **Any FF** | Fabrication and/or Falsification | 7.0  (4.2, 9.9) | 9.8  (6.6, 12.9) | 7.8  (0, 10.8) | 8.7  (6.5, 10.9) | 8.0  (5.0, 11.0) |

*Scores range from 1=never to 7=always; Prevalence is based on a Likert score of 5, 6 or 7* *among respondents that deemed the QRP at issue applicable;**Mean scores of individual QRPs only consider respondents that deemed the QRP at issue applicable.*
