# Supplementary material for: Prevalence of questionable research practices, research misconduct and their potential explanatory factors: A survey among academic researchers in The Netherlands
Source: PLoS One. 2022 Feb 16;17(2):e0263023. doi: 10.1371/journal.pone.0263023 (PMC8849616; doi:10.1371/journal.pone.0263023)
Supplement: S4 Table — (DOCX) [file pone.0263023.s007.docx]

# S4 Table. Correlation matrix of the z scores of the principal component analysis of the explanatory factor scales

| **Explanatory factor scale** | **Scientific norms** | **Peer norms** | **Work pressure** | **Publication**  **pressure** | **Funding pressure** | **Competitiveness** | **Likelihood of detection (collaborators)** | **Likelihood of detection (reviewers)** | **Mentoring** | **Mentoring (survival)** | **Mentoring (responsible)** | **Organizational justice** | **Organizational justice (distributional)** | **Organizational justice (procedural)** |
| --- | --- | --- | --- | --- | --- | --- | --- | --- | --- | --- | --- | --- | --- | --- |
| **Scientific norms** | 1.00 |  |  |  |  |  |  |  |  |  |  |  |  |  |
| **Peer norms** | 0.12 | 1.00 |  |  |  |  |  |  |  |  |  |  |  |  |
| **Work pressure** | 0.12 | -0.21 | 1.00 |  |  |  |  |  |  |  |  |  |  |  |
| **Publication pressure** | 0.09 | -0.29 | 0.39 | 1.00 |  |  |  |  |  |  |  |  |  |  |
| **Funding pressure** | 0.09 | -0.17 | 0.31 | 0.38 | 1.00 |  |  |  |  |  |  |  |  |  |
| **Competitiveness** | 0.06 | -0.41 | 0.28 | 0.44 | 0.27 | 1.00 |  |  |  |  |  |  |  |  |
| **Likelihood of detection (collaborators)** | 0.04 | 0.11 | -0.03 | -0.03 | -0.01 | -0.04 | 1.00 |  |  |  |  |  |  |  |
| **Likelihood of detection (reviewers)** | 0.07 | 0.06 | 0.02 | 0.01 | 0.06 | -0.02 | 0.46 | 1.00 |  |  |  |  |  |  |
| **Mentoring** | -0.04 | 0.23 | -0.16 | -0.16 | -0.04 | -0.29 | 0.11 | 0.06 | 1.00 |  |  |  |  |  |
| **Mentoring (survival)** | -0.04 | 0.17 | -0.11 | -0.12 | 0 | -0.24 | 0.08 | 0.06 | 0.91 | 1.00 |  |  |  |  |
| **Mentoring (responsible)** | -0.03 | 0.25 | -0.19 | -0.18 | -0.08 | -0.29 | 0.12 | 0.04 | 0.92 | 0.68 | 1.00 |  |  |  |
| **Organizational justice** | 0.02 | 0.43 | -0.33 | -0.39 | -0.31 | -0.43 | 0.08 | 0.04 | 0.35 | 0.30 | 0.33 | 1.00 |  |  |
| **Organizational justice (distributional)** | 0.02 | 0.42 | -0.33 | -0.39 | -0.30 | -0.43 | 0.07 | 0.04 | 0.33 | 0.30 | 0.31 | 0.95 | 1.00 |  |
| **Organizational justice (procedural)** | 0.02 | 0.41 | -0.29 | -0.35 | -0.28 | -0.39 | 0.07 | 0.03 | 0.33 | 0.28 | 0.33 | 0.94 | 0.80 | 1.00 |
