# Supplementary material for: Prevalence of questionable research practices, research misconduct and their potential explanatory factors: A survey among academic researchers in The Netherlands
Source: PLoS One. 2022 Feb 16;17(2):e0263023. doi: 10.1371/journal.pone.0263023 (PMC8849616; doi:10.1371/journal.pone.0263023)
Supplement: S5 Table — (DOCX) [file pone.0263023.s008.docx]

# S5 Table. Full list of the explanatory factor scales and their corresponding items

| **Explanatory factor scale**  **(CFI; RSMEA; SRMR)^#^** | **Explanatory factor scale item** | **Item-total correlations^+^** | **Factor loadings^++^** | **Reference** |
| --- | --- | --- | --- | --- |
| **Scientific norms**  **(piloted)**  **CFI : 0.92**  **RSMEA: 0.06**  **SRMR: 0.06** | 1. Researchers should evaluate research only on its merit. | 0.37 | 0.46 | [1] |
|  | 2. Researchers should judge each other’s contributions primarily on the basis of quality. | 0.49 | 0.60 | [2] |
|  | 3. The acceptance or rejection of claims entering the scholarly domain should be independent of the personal or social characteristics of researchers. | 0.36 | 0.46 | [3] |
|  | 4. Researchers should consider all new evidence, hypotheses, theories, and innovations even those that challenge or contradict their own work. | 0.39 | 0.48 | [1] |
|  | 5. Researchers should be motivated by the desire for knowledge and discovery, and not by the possibility of personal gain. | 0.40 | 0.46 | [1] |
|  | 6. Researchers should be clear about what data their work is based on and how results were achieved. | 0.39 | 0.46 | [4] |
|  | 7. Researchers contributions should never be accepted without careful scrutiny. | 0.40 | 0.52 | [3] |
|  | 8. Researchers should put their work in the public domain to be read and used freely by other researchers and the general public. (new item) | 0.26 | 0.31 |  |
|  | 9. Researchers should derive satisfaction from the mere act of doing research. (new item) | 0.27 | 0.30 |  |
| **Peer Norms**  **(piloted)**  **CFI : 0.97**  **RSMEA: 0.07**  **SRMR: 0.06** | 10. Researchers should evaluate research only on its merit. | 0.54 | 0.59 | [1] |
|  | 11. Researchers should judge each other’s contributions primarily on the basis of quality. | 0.63 | 0.69 | [2] |
|  | 12. The acceptance or rejection of claims entering the scholarly domain should be independent of the personal or social characteristics of researchers. | 0.55 | 0.61 | [3] |
|  | 13. Researchers should consider all new evidence, hypotheses, theories, and innovations even those that challenge or contradict their own work. | 0.63 | 0.70 | [1] |
|  | 14. Researchers should be motivated by the desire for knowledge and discovery, and not by the possibility of personal gain. | 0.62 | 0.68 | [1] |
|  | 15. Researchers should be clear about what data their work is based on and how results were achieved. | 0.60 | 0.65 | [4] |
|  | 16. Researchers contributions should never be accepted without careful scrutiny. | 0.54 | 0.59 | [3] |
|  | 17. Researchers should put their work in the public domain to be read and used freely by other researchers and the general public. (new item) | 0.44 | 0.48 |  |
|  | 18. Researchers should derive satisfaction from the mere act of doing research.. (new item) | 0.46 | 0.50 |  |
| **Perceived work pressure (shortened)**  **CFI : 1.00 (saturated model)**  **RSMEA: 0.00**  **SRMR: 0.00** | 19. How often does it occur that you have enough time to do all the tasks demanded of you? *(adapted item)* | 0.54 | 0.59 | [5] |
|  | 20. How often are you assigned too much work to do in a limited time? (adapted item) | 0.75 | 0.97 | [5] |
|  | 21. How often does an excess of work prevent you from having time to rest? (adapted item) | 0.62 | 0.71 | [5] |
| **Publication pressure**  **CFI : 0.99**  **RSMEA: 0.06**  **SRMR: 0.04** | 22. I feel pressure to publish. (new item) | 0.56 | 0.65 |  |
|  | 23. I experience stress at the thought of my colleagues’ assessment of my publications output. | 0.68 | 0.79 | [6] |
|  | 24. I have the feeling that my colleagues judge me mainly on the basis of my publications. (adapted item) | 0.56 | 0.65 | [6] |
|  | 25. Publication pressure harms my ability to do good research. (new item) | 0.67 | 0.74 |  |
|  | 26. The current publication climate puts pressure on relationships with fellow-researcher. | 0.58 | 0.65 | [6] |
|  | 27. Publication pressure sometimes leads me to cut corners. (adapted item) | 0.28 | 0.31 | [6] |
| **Pressure due to dependence on funding**  **CFI: 0.98**  **RSMEA: 0.06**  **SRMR: 0.04** | 28. Judgements of my academic performance are independent of my successful grant applications. (new item) | 0.22 | 0.24 |  |
|  | 29. My job security depends strongly on research grants I receive. (new item) | 0.51 | 0.60 |  |
|  | 30. My prospects for promotion depend on me obtaining funding. (new item) | 0.59 | 0.68 |  |
|  | 31. The continuation of my research depends on obtaining my own funding. (new item) | 0.65 | 0.78 |  |
|  | 32. I would be able to do my research without obtaining my own funding. (new item) | 0.46 | 0.50 |  |
|  | 33. Obtaining my own research funding is crucial for my academic career. (new item) | 0.62 | 0.75 |  |
| **Mentoring (survival)**  **CFI: 1.00**  **RSMEA: 0.05**  **SRMR: 0.03** | 34. How often has your most important mentor provided you with help in learning the art of survival in your field? (adapted item) | 0.72 | 0.80 | [2] |
|  | 35. How often has your most important mentor helped you in developing professional relationships with others in your field? (adapted item) | 0.72 | 0.80 | [2] |
|  | 36. How often has your most important mentor provided you with guidance in writing grant and contract proposals? (adapted item) | 0.61 | 0.69 | [2] |
|  | 37. How often has your most important mentor coached you in career advancement? (new item) | 0.72 | 0.86 |  |
|  | 38. How often has your most important mentor given you guidance on how to seize career opportunities? (new item) | 0.72 | 0.89 |  |
|  | 39. How often has your most important mentor advised you on how to get your research published? (new item) | 0.70 | 0.64 |  |
| **Competitiveness of research field**  **(piloted)**  **CFI: 0.82**  **RSMEA: 0.17**  **SRMR: 0.12** | 40. My field functions largely as a community of researchers. | 0.27 | 0.26 | [7] |
|  | 41. Many researchers in my field are afraid of being scooped by their peers. | 0.43 | 0.61 | [7] |
|  | 42. Many researchers in my field are unhappy when their peers obtain a major award or recognition. | 0.50 | 0.64 | [7] |
|  | 43. Rivalry between researchers is common in my field. | 0.53 | 0.75 | [7] |
|  | 44. Researchers in my field working on similar topics are inclined to collaborate with each other. | 0.45 | 0.45 | [7] |
|  | 45. Most researchers in my field consider their own work to be part of a larger collaborative effort. | 0.42 | 0.42 | [7] |
| **Mentoring (Responsible) (shortened)**  **CFI: 1.00**  **RSMEA: 0.04**  **SRMR: 0.03** | 46. How often has your most important mentor helped you in presenting the limitations of your research? (new item) | 0.72 | 0.78 |  |
|  | 47. How often has your most important mentor given you feedback on how to select the most robust research methods? (new item) | 0.74 | 0.84 |  |
|  | 48. How often has your most important mentor advised you on making your work as transparent as possible? (new item) | 0.66 | 0.77 |  |
|  | 49. How often has your most important mentor coached you on how to deal with conflicts of interest in your work? (new item) | 0.68 | 0.70 |  |
|  | 50. How often has your most important mentor provided you with insights in the ethical aspects of a research design? (new item) | 0.72 | 0.83 |  |
|  | 51. How often has your most important mentor provided you with guidance on good research practices? (adapted item) | 0.74 | 0.83 | [2] |
| **Distributional organizational justice^*^**  ***(piloted)***  **CFI: 0.99**  **RSMEA: 0.05**  **SRMR: 0.04** | 52. Resource allocation at my department is fair. (adapted item) | 0.70 | 0.74 | [8] |
|  | 53. The allocation of tasks at my department is biased.. (new item) | 0.62 | 0.67 |  |
|  | 54. Tenure decisions at my department are often biased. (new item) | 0.64 | 0.68 |  |
|  | 55. Decisions about promotion at my department are reasonable. (new item) | 0.63 | 0.67 |  |
|  | 56. The management at my department makes reasonable decisions. (new item) | 0.76 | 0.81 |  |
|  | 57. The assessment of my academic performance is fair. (new item) | 0.62 | 0.66 |  |
| **Procedural organizational justice^*^**  **(piloted)**  **CFI: 0.99**  **RSMEA: 0.04**  **SRMR: 0.03** | 58. The process of allocating resources at my department is poorly managed. (adapted item) | 0.63 | 0.61 | [8] |
|  | 59. The process of allocating tasks at my department is ethical. (adapted item) | 0.58 | 0.63 | [8] |
|  | 60. The criteria for tenure at my department are applied consistently. (new item) | 0.60 | 0.64 |  |
|  | 61. The process for promotion at my department is poor. (adapted item) | 0.58 | 0.58 | [8] |
|  | 62. The management at my department is transparent about their decisions. (new item) | 0.68 | 0.78 |  |
|  | 63. At my department, my academic performance is assessed objectively. (adapted item) | 0.70 | 0.77 | [8] |
| **Likelihood of detection by collaborators**  **(Piloted)**  **CFI: 0.93**  **RSMEA: 0.08**  **SRMR: 0.05** | How likely is it that a collaborator detects that a researcher in your field…  64. Provides insufficient supervision or mentoring to junior co-workers. (adapted item) | 0.35 | 0.42 | [9] |
|  | 65. Does not submit (or resubmit) for publication a valid negative study. (adapted item) | 0.34 | 0.42 | [9] |
|  | 66. Keeps inadequate notes of their research process in their project. (adapted item) | 0.43 | 0.55 | [9] |
|  | 67. Uses published or unpublished ideas or phrases without properly referencing the originating source. (adapted item) | 0.39 | 0.51 | [9] |
|  | 68. Unfairly reviews papers, grant applications, or colleagues applying for promotion. (adapted item) | 0.36 | 0.44 | [9] |
|  | 69. Fabricates data in his/her research. (adapted item) | 0.39 | 0.51 | [9] |
| **Likelihood of detection by reviewers**  **(Piloted)**  ***(Items are adapted to likelihood of detection format)***  **CFI: 0.99**  **RSMEA: 0.06**  **SRMR: 0.04** | How likely is it that a reviewer detects that a researcher in your field …  70. Draws conclusions that were not sufficiently substantiated by his/her study. (adapted item) | 0.61 | 0.68 | [10] |
|  | 71. Chooses an inadequate research design or uses evidently unsuitable measurement instruments for his/her study. (adapted item) | 0.67 | 0.75 | [10] |
|  | 72. Gives insufficient attention to the equipment, skills or expertise essential to perform his/her study. (adapted item) | 0.53 | 0.57 | [10] |
|  | 73. Fails to report clearly relevant details of the study method. (adapted item) | 0.66 | 0.74 | [10] |
|  | 74. Insufficiently reports study flaws and limitations. (adapted item) | 0.69 | 0.79 | [10] |
|  | 75. Selectively cites references to enhance his/her own findings or convictions. (adapted item) | 0.44 | 0.47 | [10] |

**Two subscales (distributional and procedural organizational justice) were merged due to high correlation; Table S4 shows the correlation of all the explanatory factor scales; ^+^ Corrected item-total correlations per scale for each exploratory factor was averaged over the 50 imputed data sets in the NSRI;* ^++^*item factor loadings based on confirmatory one-factor model; ^#^* *CFI: Comparative Fit Index ; RMSEA: Root Mean Square Error of Approximation; SRMR: Standardised Root Mean Square Residual*

References

1. Merton RK. The sociology of science: theoretical and empirical investigations. University of Chicago Press; 1973.

2. Anderson MS, Martinson BC, De Vries R. Normative dissonance in science: results from a national survey of U.S. scientists. J Empir Res Hum Res Ethics. 2007;2(4):3-14.

3. Mitroff II. The subjective side of science: a philosophical inquiry into the psychology of the Apollo Moon Scientists. Elsevier Scientific Publishing Company; 1974.

4. KNAW, NFU, NOW, TO2-federatie, Vereniging Hogescholen, VSNU. Netherlands code of conduct for research integrity. Report. The Netherlands: 2018.

5. Navarro MLA, Mas MB, Jiménez AML. Working conditions, burnout and stress symptoms in university professors: Validating a structural model of the mediating effect of perceived personal competence. Span J Psychol. 2010;13(1):284-96.

6. Haven TL, de Goede MEE, Tijdink JK, Oort FJ. Personally perceived publication pressure: revising the Publication Pressure Questionnaire (PPQ) by using work stress models. Res Integr Peer Rev. 2019;4:7.

7. Anderson MS, Ronning EA, De Vries R, Martinson BC. Extending the Mertonian norms: scientists’ subscription to norms of research. J Higher Educ. 2010;81(3):366-93.

8. Martinson BC, Crain AL, De Vries R, Anderson MS. The importance of organizational justice in ensuring research integrity. J Empir Res Hum Res Ethics. 2010;5(3):67-83.

9. Haven T, Tijdink J, Pasman, HR et al. Researchers’ perceptions of research misbehaviours: a mixed methods study among academic researchers in Amsterdam. Res Integr Peer Rev. 2019;4(25).

10. Bouter LM, Tijdink JK, Axelsen N, Martinson BC, ter Riet G. Ranking major and minor research misbehaviors: results from a survey among participants of four World Conferences on Research Integrity. Res Integr Peer Rev. 2016;1:17.
