# Supplementary material for: Prevalence of questionable research practices, research misconduct and their potential explanatory factors: A survey among academic researchers in The Netherlands
Source: PLoS One. 2022 Feb 16;17(2):e0263023. doi: 10.1371/journal.pone.0263023 (PMC8849616; doi:10.1371/journal.pone.0263023)
Supplement: S6 Table — (DOCX) [file pone.0263023.s009.docx]

| \| **S6. National Survey on Research Integrity Questionnaire** \| \| --- \| |
| --- | --- |

| \| Informed Consent  I understand the nature of the study and I am participating voluntarily. I understand that I can stop my participation at any time. Should I do so, my e-mail and IP address will immediately and permanently be deleted. Should I choose to complete the survey, my e-mail address and IP address will also be permanently deleted after sending. I understand there is no way to retrieve my survey once completed due to the strict privacy and anonymity arrangements of this study.  I agree to participate in this survey. \| \| --- \| |
| --- | --- |
| \| 1 \| Yes \| \| --- \| --- \| \| 2 \| No \| |

| \| No participation \| \| --- \| |
| --- | --- |
| \| We realize that you prefer not to participate in this survey. Surveys may suffer from non-response that is non-random. To help us get a clearer picture of the type of persons who prefer not to participate, we would like to collect information on their academic rank, gender, and discipline only. Would you be willing to share that information with us? It will only take a minute. \| \| --- \| |
| \| 1 \| Yes \| \| \| --- \| --- \| --- \| \| 2 \| No \|  \| |

| \| Demographics - research \| \| --- \| |
| --- | --- |
| \| Do you spend, on average, at least 8 hours a week doing research at a Dutch university or university medical center or in affiliation to one?  This may include supervision of research by Masters or PhD students. \| \| --- \| |
| \| 1 \| Yes \| \| \| --- \| --- \| --- \| \| 2 \| No \|  \| |

| \| Main Disciplinary Field \| \| --- \| |
| --- | --- |
| \| Which of the following is your main disciplinary field of research? \| \| --- \| |
| \| Select only one \| \| --- \| |
|  |
| \| 1 \| Social and Behavioral Sciences \| \| --- \| --- \| \| 2 \| Arts and Humanities \| \| 3 \| Engineering and Natural Sciences \| \| 4 \| Biomedical and Life Sciences \| |

| \| Academic Rank \| \| --- \| |
| --- | --- |
| \| Which academic rank do you belong to? \| \| --- \| |
| \| Select only one  Clarification: Full professor includes honorary, extraordinary and sponsored professorships \| \| --- \| |
|  |
| \| 1 \| PhD student or junior researcher \| \| \| --- \| --- \| --- \| \| 2 \| Postdoctoral researcher, Assistant Professor, Physician Researcher or Lecturer Researcher \| \| \| 3 \| Associate Professor or Full Professor \| \| \| 4 \| None of the above \|  \| |

| \| Gender \| \| --- \| |
| --- | --- |
| \| What is your gender? \| \| --- \| |
| \| 1 \| Female \| \| --- \| --- \| \| 2 \| Male \| \| 3 \| Other / Do not wish to disclose \| |

| \| Engage in research \| \| --- \| |
| --- | --- |
| \| Were you engaged in empirical research in the last three years? \| \| --- \| |
| \| Clarification: Empirical research refers to research in which quantitative or qualitative data is collected and/or analysed. Non-empirical research refers to research such as hermeneutic reflection, development of theories, models or codes, and academic design activities like in art or architecture. \| \| --- \| |
|  |
| \| 1 \| Yes \| \| --- \| --- \| \| 2 \| No \| |

| \| Factors influencing Research Practices \| \| --- \| |
| --- | --- |
| \| This section includes a number of statements covering a broad range of issues that may help or hinder responsible conduct of research.  You will now be presented with a number of statements and be asked to rate them. \| \| --- \| |

| \| **Work Pressure**  In this section, we present you with items on perceived work pressure, publication pressure, pressure due to funding, competitiveness in your field and mentoring.  For each statement, please indicate your most appropriate answer.  How often does it occur that you have enough time to do all the tasks demanded of you? \| \| --- \| |
| --- | --- |
| \| Never \| 1 2 3 4 5 6 7 \| Always \| \| --- \| --- \| --- \| |

| \| **Work Pressure**  How often are you assigned too much work to do in a limited time? \| \| --- \| |
| --- | --- |
| \| Never \| 1 2 3 4 5 6 7 \| Always \| \| --- \| --- \| --- \| |
|  |

| \| **Work Pressure**  How often does an excess of work prevent you from having time to rest? \| \| --- \| |
| --- | --- |
| \| Never \| 1 2 3 4 5 6 7 \| Always \| \| --- \| --- \| --- \| |

| \| **Publication Pressure**  I feel pressure to publish. \| \| --- \| |
| --- | --- |
| \| Totally Disagree \| 1 2 3 4 5 6 7 \| Totally Agree \| \| --- \| --- \| --- \| |

| \| **Publication Pressure**  I experience stress at the thought of my colleagues’ assessment of my publication output. \| \| --- \| |
| --- | --- |
| \| Totally Disagree \| 1 2 3 4 5 6 7 \| Totally Agree \| \| --- \| --- \| --- \| |

| \| **Publication Pressure**  I have the feeling that my colleagues judge me mainly on the basis of my publications. \| \| --- \| |
| --- | --- |
| \| Totally Disagree \| 1 2 3 4 5 6 7 \| Totally Agree \| \| --- \| --- \| --- \| |

| \| **Publication Pressure**  Publication pressure harms my ability to do good research. \| \| --- \| |
| --- | --- |
| \| Totally Disagree \| 1 2 3 4 5 6 7 \| Totally Agree \| \| --- \| --- \| --- \| |

| \| **Publication Pressure**  The current publication climate puts pressure on relationships with fellow-researchers. \| \| --- \| |
| --- | --- |
| \| Totally Disagree \| 1 2 3 4 5 6 7 \| Totally Agree \| \| --- \| --- \| --- \| |

| \| **Publication Pressure**  Publication pressure sometimes leads me to cut corners. \| \| --- \| |
| --- | --- |
| \| *Clarification: Cutting corners refers to engaging in questionable research practices to safe time or to get spectacular results.* \| \| --- \| |
|  |
| \| Totally Disagree \| 1 2 3 4 5 6 7 \| Totally Agree \| \| --- \| --- \| --- \| |

| \| **Pressure Due to Funding**  Judgements of my academic performance are independent of my successful grant applications. \| \| --- \| |
| --- | --- |
| \| Totally Disagree \| 1 2 3 4 5 6 7 \| Totally Agree \| \| --- \| --- \| --- \| |

| Not applicable |
| --- |

| \| **Pressure Due to Funding**  My job security depends strongly on research grants I receive. \| \| --- \| |
| --- | --- |
| \| Totally Disagree \| 1 2 3 4 5 6 7 \| Totally Agree \| \| --- \| --- \| --- \| |
| \| Not applicable \| \| --- \| |
|  |
| \| **Pressure Due to Funding**  My prospects for promotion depend on me obtaining funding. \| \| --- \| |
| \| Totally Disagree \| 1 2 3 4 5 6 7 \| Totally Agree \| \| --- \| --- \| --- \| |
| \| Not applicable \| \| --- \| |
| \| **Pressure Due to Funding**  The continuation of my research depends on obtaining my own funding. \| \| --- \| |
| \| Totally Disagree \| 1 2 3 4 5 6 7 \| Totally Agree \| \| --- \| --- \| --- \| |
| \| Not applicable \| \| --- \|  \| **Pressure Due to Funding**  I would be able to do my research without obtaining my own funding. \| \| --- \| |
| \| Totally Disagree \| 1 2 3 4 5 6 7 \| Totally Agree \| \| --- \| --- \| --- \| |
| \| Not applicable \| \| --- \|  \| **Pressure Due to Funding**  Obtaining my own research funding is crucial for my academic career. \| \| --- \| |
| \| Totally Disagree \| 1 2 3 4 5 6 7 \| Totally Agree \| \| --- \| --- \| --- \| |
| \| Not applicable \| \| --- \| |

| \| **Survival Mentoring**  How often has your most important mentor provided you with help in learning the art of survival in your field? \| \| --- \| |
| --- | --- |
| \| *Clarification: A mentor may be a person from the past or present who may be your supervisor, colleague or peer to whom you looked to for guidance in your academic career.* \| \| --- \| |
|  |
| \| Never \| 1 2 3 4 5 6 7 \| Always \| \| --- \| --- \| --- \| |

| \| **Survival Mentoring**  How often has your most important mentor helped you in developing professional relationships with others in your field? \| \| --- \| |
| --- | --- |
| \| Never \| 1 2 3 4 5 6 7 \| Always \| \| --- \| --- \| --- \| |

| \| **Survival Mentoring**  How often has your most important mentor provided you with guidance in writing grant and contract proposals? \| \| --- \| |
| --- | --- |
| \| Never \| 1 2 3 4 5 6 7 \| Always \| \| --- \| --- \| --- \| |

| \| **Survival Mentoring**  How often has your most important mentor coached you in career advancement? \| \| --- \| |
| --- | --- |
| \| Never \| 1 2 3 4 5 6 7 \| Always \| \| --- \| --- \| --- \| |

| \| **Survival Mentoring**  How often has your most important mentor given you guidance on how to seize career opportunities? \| \| --- \| |
| --- | --- |
| \| Never \| 1 2 3 4 5 6 7 \| Always \| \| --- \| --- \| --- \| |

| \| **Survival Mentoring**  How often has your most important mentor advised you on how to get your research published? \| \| --- \| |
| --- | --- |
| \| Never \| 1 2 3 4 5 6 7 \| Always \| \| --- \| --- \| --- \| |

| \| **Competitiveness of your field**  My field functions largely as a community of researchers. \| \| --- \| |
| --- | --- |
| \| Totally Disagree \| 1 2 3 4 5 6 7 \| Totally Agree \| \| --- \| --- \| --- \| |

| \| **Competitiveness of your field**  Many researchers in my field are afraid of being scooped by their peers. \| \| --- \| |
| --- | --- |
| \| Clarification: Scooping refers to using in grant proposals and/or papers ideas or materials of your peers without their approval or proper referencing. \| \| --- \| |
|  |
| \| Totally Disagree \| 1 2 3 4 5 6 7 \| Totally Agree \| \| --- \| --- \| --- \| |

| \| **Competitiveness of your field**  Many researchers in my field are unhappy when their peers obtain a major award or recognition. \| \| --- \| |
| --- | --- |
| \| Totally Disagree \| 1 2 3 4 5 6 7 \| Totally Agree \| \| --- \| --- \| --- \| |

| \| **Competitiveness of your field**  Rivalry between researchers is common in my field. \| \| --- \| |
| --- | --- |
| \| Totally Disagree \| 1 2 3 4 5 6 7 \| Totally Agree \| \| --- \| --- \| --- \| |

| \| **Competitiveness of your field**  *Congratulations, you completed 25% of the survey.*  Researchers in my field working on similar topics are inclined to collaborate with each other. \| \| --- \| |
| --- | --- |
| \| Totally Disagree \| 1 2 3 4 5 6 7 \| Totally Agree \| \| --- \| --- \| --- \| |

| \| **Competitiveness of your field**  Most researchers in my field consider their own work to be part of a larger collaborative effort. \| \| --- \| |
| --- | --- |
| \| Totally Disagree \| 1 2 3 4 5 6 7 \| Totally Agree \| \| --- \| --- \| --- \| |

| \| **Responsible Mentoring**  How often has your most important mentor helped you in presenting the limitations of your research? \| \| --- \| |
| --- | --- |
| \| *Clarification: A mentor may be a person from the past or present who may be your supervisor, colleague or peer to whom you looked to for guidance in your academic career.* \| \| --- \| |
|  |
| \| Never \| 1 2 3 4 5 6 7 \| Always \| \| --- \| --- \| --- \| |

| \| **Responsible Mentoring**  How often has your most important mentor given you feedback on how to select the most robust research methods? \| \| --- \| |
| --- | --- |
| \| Never \| 1 2 3 4 5 6 7 \| Always \| \| --- \| --- \| --- \| |

| \| **Responsible Mentoring**  How often has your most important mentor advised you on making your work as transparent as possible? \| \| --- \| |
| --- | --- |
| \| Never \| 1 2 3 4 5 6 7 \| Always \| \| --- \| --- \| --- \| |

| \| **Responsible Mentoring**  How often has your most important mentor coached you on how to deal with conflicts of interest in your work? \| \| --- \| |
| --- | --- |
| \| Never \| 1 2 3 4 5 6 7 \| Always \| \| --- \| --- \| --- \| |

| \| **Responsible Mentoring**  How often has your most important mentor provided you with insights in the ethical aspects of a research design? \| \| --- \| |
| --- | --- |
| \| Never \| 1 2 3 4 5 6 7 \| Always \| \| --- \| --- \| --- \| |

| \| **Responsible Mentoring**  How often has your most important mentor provided you with guidance on good research practices? \| \| --- \| |
| --- | --- |
| \| *Clarification:Good research practices refer to behaviors undertaken to adhere to standards for high quality research within the discipline and guided by principles of honesty, transparency and independance.* \| \| --- \| |
|  |
| \| Never \| 1 2 3 4 5 6 7 \| Always \| \| --- \| --- \| --- \| |

| \| **Scientific Norms**  Scientific Norms refer to ideals a reseacher may subscribe to. For each of the following statements, please indicate the extent to which you personally feel the items **should** represent the **ideal** behavior of researchers.  Researchers **should** evaluate research only on its merit. \| \| --- \| |
| --- | --- |
| \| *Clarification: Merit refers to the value of research in terms of improving knowledge or potential societal relevance.* \| \| --- \| |
|  |
| \| Totally Disagree \| 1 2 3 4 5 6 7 \| Totally Agree \| \| --- \| --- \| --- \| |

| \| **Scientific Norms**  Researchers **should** judge each other’s contributions primarily on the basis of quality. \| \| --- \| |
| --- | --- |
| \| Totally Disagree \| 1 2 3 4 5 6 7 \| Totally Agree \| \| --- \| --- \| --- \| |

| \| **Scientific Norms**  The acceptance or rejection of claims entering the scholarly domain **should** be independent of the personal or social characteristics of researchers. \| \| --- \| |
| --- | --- |
| \| Totally Disagree \| 1 2 3 4 5 6 7 \| Totally Agree \| \| --- \| --- \| --- \| |

| \| **Scientific Norms**  Researchers **should** consider all new evidence, hypotheses, theories, and innovations even those that challenge or contradict their own work. \| \| --- \| |
| --- | --- |
| \| Totally Disagree \| 1 2 3 4 5 6 7 \| Totally Agree \| \| --- \| --- \| --- \| |

| \| **Scientific Norms**  Researchers **should be** motivated by the desire for knowledge and discovery, and not by the possibility of personal gain. \| \| --- \| |
| --- | --- |
| \| Totally Disagree \| 1 2 3 4 5 6 7 \| Totally Agree \| \| --- \| --- \| --- \| |

| \| **Scientific Norms**  Researchers **should be** clear about what data their work is based on and how results were achieved. \| \| --- \| |
| --- | --- |
| \| Totally Disagree \| 1 2 3 4 5 6 7 \| Totally Agree \| \| --- \| --- \| --- \| |

| \| **Scientific Norms**  Researchers contributions **should** never be accepted without careful scrutiny. \| \| --- \| |
| --- | --- |
| \| Totally Disagree \| 1 2 3 4 5 6 7 \| Totally Agree \| \| --- \| --- \| --- \| |

| \| **Scientific Norms**  Researchers **should** put their work in the public domain to be read and used freely by other researchers and the general public. \| \| --- \| |
| --- | --- |
| \| Totally Disagree \| 1 2 3 4 5 6 7 \| Totally Agree \| \| --- \| --- \| --- \| |

| \| **Scientific Norms**  Researchers **should** derive satisfaction from the mere act of doing research. \| \| --- \| |
| --- | --- |
| \|  \| \| --- \| |
| \| Totally Disagree \| 1 2 3 4 5 6 7 \| Totally Agree \| \| --- \| --- \| --- \| |

| \| **Normative Behavior of Academic Peers**  Normative behavior of academic peers refer to your perception of your peers **actual** behavior. For each of the following statements below, please indicate the extent to which you feel the following items **actually represent the typical behavior of your peers**.  Researchers **actually** evaluate research only on its merit. \| \| --- \| |
| --- | --- |
| \| *Clarification: Merit refers to the value of research in terms of improving knowledge or potential societal relevance.* \| \| --- \| |
|  |
| \| Totally Disagree \| 1 2 3 4 5 6 7 \| Totally Agree \| \| --- \| --- \| --- \| |

| \| **Normative Behavior of Academic Peers**  Researchers **actually** judge each other’s contributions primarily on the basis of quality. \| \| --- \| |
| --- | --- |
| \| Totally Disagree \| 1 2 3 4 5 6 7 \| Totally Agree \| \| --- \| --- \| --- \| |

| \| **Normative Behavior of Academic Peers**  The acceptance or rejection of claims entering the scholarly domain is **actually** independent of the personal or social characteristics of researchers. \| \| --- \| |
| --- | --- |
| \| Totally Disagree \| 1 2 3 4 5 6 7 \| Totally Agree \| \| --- \| --- \| --- \| |

| \| **Normative Behavior of Academic Peers**  Researchers **actually** consider all new evidence, hypotheses, theories, and innovations even those that challenge or contradict their own work. \| \| --- \| |
| --- | --- |
| \| Totally Disagree \| 1 2 3 4 5 6 7 \| Totally Agree \| \| --- \| --- \| --- \| |

| \| **Normative Behavior of Academic Peers**  Researchers are **actually** motivated by the desire for knowledge and discovery, and not by the possibility of personal gain. \| \| --- \| |
| --- | --- |
| \| Totally Disagree \| 1 2 3 4 5 6 7 \| Totally Agree \| \| --- \| --- \| --- \| |

| \| **Normative Behavior of Academic Peers**  Researchers **actually** are clear about what data their work is based on and how results were achieved. \| \| --- \| |
| --- | --- |
| \| Totally Disagree \| 1 2 3 4 5 6 7 \| Totally Agree \| \| --- \| --- \| --- \| |

| \| **Normative Behavior of Academic Peers**  A researcher's contribution to knowledge is never **actually** accepted without careful scrutinty. \| \| --- \| |
| --- | --- |
| \| Totally Disagree \| 1 2 3 4 5 6 7 \| Totally Agree \| \| --- \| --- \| --- \| |

| \| **Normative Behavior of Academic Peers**  Researchers **actually** put their work in the public domain to be read and used by other researchers and the general public. \| \| --- \| |
| --- | --- |
| \| Totally Disagree \| 1 2 3 4 5 6 7 \| Totally Agree \| \| --- \| --- \| --- \| |

| \| **Normative Behavior of Academic Peers**  Researchers **actually** derive satisfaction from the mere act of doing research. \| \| --- \| |
| --- | --- |
| \| Totally Disagree \| 1 2 3 4 5 6 7 \| Totally Agree \| \| --- \| --- \| --- \| |

| \| In this section, we will present you with items relating to organizational justice. For each statement, please indicate the extent to which you agree with it.  **Distributional Organizational Justice**  Resource allocation at my department is fair. \| \| --- \| |
| --- | --- |
| \| Totally Disagree \| 1 2 3 4 5 6 7 \| Totally Agree \| \| --- \| --- \| --- \| |

| \| **Distributional Organizational Justice**  The allocation of tasks at my department is biased. \| \| --- \| |
| --- | --- |
| \| Totally Disagree \| 1 2 3 4 5 6 7 \| Totally Agree \| \| --- \| --- \| --- \| |

| \| **Distributional Organizational Justice**  Tenure decisions at my department are often biased. \| \| --- \| |
| --- | --- |
| \| Totally Disagree \| 1 2 3 4 5 6 7 \| Totally Agree \| \| --- \| --- \| --- \| |
|  |

| \| **Distributional Organizational Justice**  Decisions about promotion at my department are reasonable. \| \| --- \| |
| --- | --- |
| \| Totally Disagree \| 1 2 3 4 5 6 7 \| Totally Agree \| \| --- \| --- \| --- \| |

| \| **Distributional Organizational Justice**  The management at my department makes reasonable decisions. \| \| --- \| |
| --- | --- |
| \| Totally Disagree \| 1 2 3 4 5 6 7 \| Totally Agree \| \| --- \| --- \| --- \| |

| \| **Distributional Organizational Justice**  The assessment of my academic performance in the past 3 years is fair. \| \| --- \| |
| --- | --- |
| \| Totally Disagree \| 1 2 3 4 5 6 7 \| Totally Agree \| \| --- \| --- \| --- \| |

| \| **Procedural Organizational Justice**  *Congratulations, you completed 50% of the survey.*  The process of allocating resources at my department is poorly managed. \| \| --- \| |
| --- | --- |
| \| Totally Disagree \| 1 2 3 4 5 6 7 \| Totally Agree \| \| --- \| --- \| --- \| |
| \| Not applicable \| \| --- \| |

| \| **Procedural Organizational Justice**  The process of allocating tasks at my department is ethical. \| \| --- \| |
| --- | --- |
| \| Totally Disagree \| 1 2 3 4 5 6 7 \| Totally Agree \| \| --- \| --- \| --- \| |
| \| Not applicable \| \| --- \| |

| \| **Procedural Organizational Justice**  The criteria for tenure at my department are applied consistently. \| \| --- \| |
| --- | --- |
| \| Totally Disagree \| 1 2 3 4 5 6 7 \| Totally Agree \| \| --- \| --- \| --- \| |
| \| Not applicable \| \| --- \| |

| \| **Procedural Organizational Justice**  The process for promotion at my department is poor. \| \| --- \| |
| --- | --- |
| \| Totally Disagree \| 1 2 3 4 5 6 7 \| Totally Agree \| \| --- \| --- \| --- \| |
| \| Not applicable \| \| --- \| |

| \| **Procedural Organizational Justice**  The management at my department is transparent about their decisions. \| \| --- \| |
| --- | --- |
| \| Totally Disagree \| 1 2 3 4 5 6 7 \| Totally Agree \| \| --- \| --- \| --- \| |
| \| Not applicable \| \| --- \| |

| \| **Procedural Organizational Justice**  At my department, academic performances are assessed objectively. \| \| --- \| |
| --- | --- |
| \| Totally Disagree \| 1 2 3 4 5 6 7 \| Totally Agree \| \| --- \| --- \| --- \| |
| \| Not applicable \| \| --- \| |

| \| **Likelihood of QRP detection**  In this section, we present particular Questionable Research Practices (QRPs) and ask you to indicate how likely it is that these QRPs are detected by:  **Collaborators:** Defined as students, colleagues, or other academics with whom the researcher works together on one or more research projects.  **Reviewers:** Defined as academic peers who in the context of publishing the work independently assess its quality.  If the described practice does not apply to research in your field, please select 'Not applicable' \| \| --- \| |
| --- | --- |

| \| **QRP detection by collaborator**  How likely is it that a **collaborator** *detects* that a researcher in your field...  Provides insufficient supervision or mentoring to junior co-workers. \| \| --- \| |
| --- | --- |
| \| *Collaborators: Defined as students, colleagues, or other academics with whom the researcher works together on one or more research projects.* \| \| --- \| |
|  |
| \| Very Unlikely \| 1 2 3 4 5 6 7 \| Very Likely \| \| --- \| --- \| --- \| |
| \| Not applicable \| \| --- \| |

| \| **QRP detection by collaborator**  How likely is it that a **collaborator** *detects* that a researcher in your field...  Does not submit (or resubmit) for publication a valid negative study. \| \| --- \| |
| --- | --- |
| \| *Collaborators: Defined as students, colleagues, or other academics with whom the researcher works together on one or more research projects.*  *Clarification: A valid negative study may be defined as one that did not support your original study hypothesis.* \| \| --- \| |
|  |
| \| Very Unlikely \| 1 2 3 4 5 6 7 \| Very Likely \| \| --- \| --- \| --- \| |
| \| Not applicable \| \| --- \| |

| \| **QRP detection by collaborator**  How likely is it that a **collaborator** *detects* that a researcher in your field...  Keeps inadequate notes of their research process in their project. \| \| --- \| |
| --- | --- |
| \| *Collaborators: Defined as students, colleagues, or other academics with whom the researcher works together on one or more research projects.* \| \| --- \| |
|  |
| \| Very Unlikely \| 1 2 3 4 5 6 7 \| Very Likely \| \| --- \| --- \| --- \| |
| \| Not applicable \| \| --- \| |

| \| **QRP detection by collaborator**  How likely is it that a **collaborator** *detects* that a researcher in your field...  Uses published or unpublished ideas or phrases without properly referencing the originating source. \| \| --- \| |
| --- | --- |
| \| *Collaborators: Defined as students, colleagues, or other academics with whom the researcher works together on one or more research projects.* \| \| --- \| |
|  |
| \| Very Unlikely \| 1 2 3 4 5 6 7 \| Very Likely \| \| --- \| --- \| --- \| |
| \| Not applicable \| \| --- \| |

| \| **QRP detection by collaborator**  How likely is it that a **collaborator** *detects* that a researcher in your field...  Unfairly reviews papers, grant applications, or colleagues applying for promotion. \| \| --- \| |
| --- | --- |
| \| *Collaborators: Defined as students, colleagues, or other academics with whom the researcher works together on one or more research projects.* \| \| --- \| |
|  |
| \| Very Unlikely \| 1 2 3 4 5 6 7 \| Very Likely \| \| --- \| --- \| --- \| |
| \| Not applicable \| \| --- \| |

| \| **QRP detection by collaborator**  How likely is it that a **collaborator** *detects* that a researcher in your field...  Fabricates data in his/her research. \| \| --- \| |
| --- | --- |
| \| *Collaborators: Defined as students, colleagues, or other academics with whom the researcher works together on one or more research projects.* \| \| --- \| |
|  |
| \| Very Unlikely \| 1 2 3 4 5 6 7 \| Very Likely \| \| --- \| --- \| --- \| |
| \| Not applicable \| \| --- \| |

| \| **QRP detection by reviewer**  How likely is it that a **reviewer** *detects* that a researcher in your field...  Draws conclusions that were not sufficiently substantiated by his/her study. \| \| --- \| |
| --- | --- |
| \| *Reviewers: Defined as academic peers who, in the context of publishing the work independently assess its quality.* \| \| --- \| |
|  |
| \| Very Unlikely \| 1 2 3 4 5 6 7 \| Very Likely \| \| --- \| --- \| --- \| |
| \| Not applicable \| \| --- \| |

| \| **QRP detection by reviewer**  How likely is it that a **reviewer** *detects* that a researcher in your field...  Chooses an inadequate research design or uses evidently unsuitable measurement instruments for his/her study. \| \| --- \| |
| --- | --- |
| \| *Reviewers: Defined as academic peers who, in the context of publishing the work independently assess its quality.* \| \| --- \| |
|  |
| \| Very Unlikely \| 1 2 3 4 5 6 7 \| Very Likely \| \| --- \| --- \| --- \| |
| \| Not applicable \| \| --- \| |

| \| **QRP detection by reviewer**  How likely is it that a **reviewer** *detects* that a researcher in your field...  Gives insufficient attention to the equipment, skills or expertise essential to perform his/her study. \| \| --- \| |
| --- | --- |
| \| *Reviewers: Defined as academic peers who, in the context of publishing the work independently assess its quality.* \| \| --- \| |
|  |
| \| Very Unlikely \| 1 2 3 4 5 6 7 \| Very Likely \| \| --- \| --- \| --- \| |
| \| Not applicable \| \| --- \| |

| \| **QRP detection by reviewer**  How likely is it that a **reviewer** **detects** that a researcher in your field...  Fails to report clearly relevant details of the study method. \| \| --- \| |
| --- | --- |
| \| *Reviewers: Defined as academic peers who, in the context of publishing the work independently assess its quality.* \| \| --- \| |
|  |
| \| Very Unlikely \| 1 2 3 4 5 6 7 \| Very Likely \| \| --- \| --- \| --- \| |
| \| Not applicable \| \| --- \| |

| \| **QRP detection by reviewer**  How likely is it that a **reviewer** *detects* that a researcher in your field...  Insufficiently reports study flaws and limitations. \| \| --- \| |
| --- | --- |
| \| *Reviewers: Defined as academic peers who, in the context of publishing the work independently assess its quality.* \| \| --- \| |
|  |
| \| Very Unlikely \| 1 2 3 4 5 6 7 \| Very Likely \| \| --- \| --- \| --- \| |
| \| Not applicable \| \| --- \| |

| \| **QRP detection by reviewer**  How likely is it that a **reviewer** *detects* that a researcher in your field...  Selectively cites references to enhance his/her own findings or convictions. \| \| --- \| |
| --- | --- |
| \| *Reviewers: Defined as academic peers who, in the context of publishing the work independently assess its quality.* \| \| --- \| |
|  |
| \| Very Unlikely \| 1 2 3 4 5 6 7 \| Very Likely \| \| --- \| --- \| --- \| |
| \| Not applicable \| \| --- \| |

| \| **Research practices in your discipline**  Please specify how often you engage in the research practices listed on the following screens.  If the research practice does not apply to you, please select 'Not applicable'. \| \| --- \| |
| --- | --- |

| \| Research Practices \| \| --- \| |
| --- | --- |
| \| *In the last three years,* I disclosed who funded my studies and all my relevant financial and non-financial interests in my publications. \| \| --- \| |
| \| Never \| 1 2 3 4 5 6 7 \| Always \| \| --- \| --- \| --- \| |
| \| Not applicable \| \| --- \| |

| \| Research Practices \| \| --- \| |
| --- | --- |
| \| *In the last three years*, I took steps to correct errors in my published work whenever I and/or peers provided valid reasons for such a correction. \| \| --- \| |
| \| Never \| 1 2 3 4 5 6 7 \| Always \| \| --- \| --- \| --- \| |
| \| Not applicable \| \| --- \| |

| \| **Research practices in your discipline**  *In the last three years,* I gave *insufficient* attention to the equipment, skills or expertise essential to perform my studies. \| \| --- \| |
| --- | --- |
| \| Never \| 1 2 3 4 5 6 7 \| Always \| \| --- \| --- \| --- \| |
| \| Not applicable \| \| --- \| |

| \| Research Practices \| \| --- \| |
| --- | --- |
| \| *In the last three years,* the allocation and ordering of authorships in my publications, were fair and in line with the standards of my discipline. \| \| --- \| |
| \| Clarification: Fair allocation refers to inclusion of all authors who made a genuine intellectual contribution to at least one of the following elements: the design of the research, the acquisition of data, its analysis or the interpretation of findings. \| \| --- \| |
|  |
| \| Never \| 1 2 3 4 5 6 7 \| Always \| \| --- \| --- \| --- \| |
| \| Not applicable \| \| --- \| |

| \| Research Practices \| \| --- \| |
| --- | --- |
| \| *Congratulations, you completed 75% of the survey.*  *In the last three years,* I *insufficiently* supervised or mentored junior co-workers. \| \| --- \| |
| \| Never \| 1 2 3 4 5 6 7 \| Always \| \| --- \| --- \| --- \| |
| \| Not applicable \| \| --- \| |

| \| Research Practices \| \| --- \| |
| --- | --- |
| \| *In the last three years,* I contributed, where appropriate, to making my research data findable, accessible, interoperable and reusable in accordance with the FAIR principles. \| \| --- \| |
| \| Clarification: For some types of research this means providing detailed descriptions of where source material can be found (complete with relevant file numbers, page numbers, etc.) \| \| --- \| |
|  |
| \| Never \| 1 2 3 4 5 6 7 \| Always \| \| --- \| --- \| --- \| |
| \| Not applicable \| \| --- \| |

| \| Research Practices \| \| --- \| |
| --- | --- |
| \| *In the last three years*, I chose inadequate research designs or used evidently unsuitable measurement instruments for my studies. \| \| --- \| |
| \| Clarification: An "inadequate research design" or "measurement instrument" may also refer to knowingly choosing a research approach or theory that is clearly inadequate to answer the overall study question \| \| --- \| |
|  |
| \| Never \| 1 2 3 4 5 6 7 \| Always \| \| --- \| --- \| --- \| |
| \| Not applicable \| \| --- \| |

| \| Research Practices \| \| --- \| |
| --- | --- |
| \| *In the last three years,* I unfairly reviewed manuscripts, grant applications or colleagues applying for promotion. \| \| --- \| |
| \| Never \| 1 2 3 4 5 6 7 \| Always \| \| --- \| --- \| --- \| |
| \| Not applicable \| \| --- \| |

| \| Research Practices \| \| --- \| |
| --- | --- |
| \| *In the last three years,* I kept a comprehensive record of my research decisions throughout my studies. \| \| --- \| |
| \| Never \| 1 2 3 4 5 6 7 \| Always \| \| --- \| --- \| --- \| |
| \| Not applicable \| \| --- \| |

| \| Research Practices \| \| --- \| |
| --- | --- |
| \| *In the last three years,* I drew conclusions that were not sufficiently substantiated by my studies. \| \| --- \| |
| \| Clarification: This refers to instances when you let your own convictions guide the conclusions of your study more than is warranted by the data \| \| --- \| |
|  |
| \| Never \| 1 2 3 4 5 6 7 \| Always \| \| --- \| --- \| --- \| |
| \| Not applicable \| \| --- \| |

| \| Research Practices \| \| --- \| |
| --- | --- |
| \| *In the last three years,* I used published or unpublished ideas or phrases from others without properly referencing its source. \| \| --- \| |
| \| Never \| 1 2 3 4 5 6 7 \| Always \| \| --- \| --- \| --- \| |
| \| Not applicable \| \| --- \| |

| \| Research Practices \| \| --- \| |
| --- | --- |
| \| *In the last three years*, I pre-registered my study protocols in line with open science practices. \| \| --- \| |
| \| Never \| 1 2 3 4 5 6 7 \| Always \| \| --- \| --- \| --- \| |
| \| Not applicable \| \| --- \| |

| \| Research Practices \| \| --- \| |
| --- | --- |
| \| *In the last three years,* I kept *inadequate* notes of my research process in a project. \| \| --- \| |
| \| Never \| 1 2 3 4 5 6 7 \| Always \| \| --- \| --- \| --- \| |
| \| Not applicable \| \| --- \| |

| \| Research Practices \| \| --- \| |
| --- | --- |
| \| *In the last three years,* I did not mention clearly important details of my study method in my publications. \| \| --- \| |
| \| Never \| 1 2 3 4 5 6 7 \| Always \| \| --- \| --- \| --- \| |
| \| Not applicable \| \| --- \| |

| \| Research Practices \| \| --- \| |
| --- | --- |
| \| *In the last three years*, I managed my research data carefully by storing both the raw and processed versions for a period appropriate to my discipline and methodology used. \| \| --- \| |
| \| Never \| 1 2 3 4 5 6 7 \| Always \| \| --- \| --- \| --- \| |
| \| Not applicable \| \| --- \| |

| \| Research Practices \| \| --- \| |
| --- | --- |
| \| *In the last three years*, my research was published under open access conditions. \| \| --- \| |
| \| Clarification: 'Open access' publication refers to publication where there are no financial, legal or technical barriers to accessing it \| \| --- \| |
|  |
| \| Never \| 1 2 3 4 5 6 7 \| Always \| \| --- \| --- \| --- \| |
| \| Not applicable \| \| --- \| |

| \| Research Practices \| \| --- \| |
| --- | --- |
| \| *In the last three years,* when making use of other people’s ideas, procedures, results and text in my publications, I cited the source accurately in accordance with the standards of my discipline. \| \| --- \| |
| \| Never \| 1 2 3 4 5 6 7 \| Always \| \| --- \| --- \| --- \| |
| \| Not applicable \| \| --- \| |

| \| Research Practices \| \| --- \| |
| --- | --- |
| \| *In the last three years*, I chose not to submit or resubmit valid negative studies for publication. \| \| --- \| |
| \| *Clarification: A valid negative study may be defined as one that did not support your original study hypothesis* \| \| --- \| |
|  |
| \| Never \| 1 2 3 4 5 6 7 \| Always \| \| --- \| --- \| --- \| |
| \| Not applicable \| \| --- \| |

| \| Research Practices \| \| --- \| |
| --- | --- |
| \| *In the last three years*, I fully disclosed and made accessible on open science platforms my underlying data, computer codes, or syntaxes used in my research. \| \| --- \| |
| \| Never \| 1 2 3 4 5 6 7 \| Always \| \| --- \| --- \| --- \| |
| \| Not applicable \| \| --- \| |

| \| Research Practices \| \| --- \| |
| --- | --- |
| \| *In the last three years*, I insufficiently mentioned study flaws and limitations in my publications. \| \| --- \| |
| \| Never \| 1 2 3 4 5 6 7 \| Always \| \| --- \| --- \| --- \| |
| \| Not applicable \| \| --- \| |

| \| Research Practices \| \| --- \| |
| --- | --- |
| \| *In the last three years,* before releasing results of my research, I meticulously checked my work to avoid errors and biases. \| \| --- \| |
| \| Never \| 1 2 3 4 5 6 7 \| Always \| \| --- \| --- \| --- \| |
| \| Not applicable \| \| --- \| |

| \| Research Practices \| \| --- \| |
| --- | --- |
| \| *In the last three years,* I selectively cited references to enhance my own findings or convictions. \| \| --- \| |
| \| *Clarification: This also refers to intentionally excluding references that might undermine your theory or the argument you want to make* \| \| --- \| |
|  |
| \| Never \| 1 2 3 4 5 6 7 \| Always \| \| --- \| --- \| --- \| |
| \| Not applicable \| \| --- \| |

| \| Randomized Response \| \| --- \| |
| --- | --- |
| \| The following questions require the use of the Randomized Response Device.  If you like to learn more on how the Randomized Response device protects your true answer, this video which takes a few minutes explains how.  You could skip this video by clicking on the next button. \| \| --- \| |
| \| Randomized Response \| \| --- \| |
| \| Click below for a short demonstration on how to use this device to correctly select your answer.    This video is without sound.  You could skip this video by clicking on the next button. \| \| --- \| |

| \| Randomized Response \| \| --- \| |
| --- | --- |
| \| You will now be presented with two try-out questions to help you understand how to select your answers using the randomized response device.  You could skip these questions if you like.  Have you had a cup of coffee today? \| \| --- \| |
| \| 1. Click on the 'start' button  2. The circle and triangle will start alternate  3. Click on the 'stop' button when you are ready to answer  4. Choose the symbol that represents your answer \| \| --- \| |
|  |
| \| 1 \| Circle \| \| --- \| --- \| \| 2 \| Triangle \| |

| \| Randomized Response \| \| --- \| |
| --- | --- |
| \| Have you ignored a red traffic light in the previous 12 months? \| \| --- \| |
| \| 1. Click on the 'start' button  2. The circle and triangle will start alternate  3. Click on the 'stop' button when you are ready to answer  4. Choose the symbol that represents your answer \| \| --- \| |
|  |
| \| 1 \| Circle \| \| --- \| --- \| \| 2 \| Triangle \| |

| \| Please answer the two questions on the following screens using the Randomized Response device. \| \| --- \| |
| --- | --- |

| \| Randomized Response \| \| --- \| |
| --- | --- |
| \| In the last three years, I fabricated data in my research. \| \| --- \| |
| \| *Clarification: Fabrication is making up data or results and recording or reporting them as real.*    1. Click on the 'start' button  2. The circle and triangle will start alternate  3. Click on the 'stop' button when you are ready to answer  4. Choose the symbol that represents your answer \| \| --- \| |
|  |
| \| 1 \| Circle \| \| --- \| --- \| \| 2 \| Triangle \| |
|  |

| \| Randomized Response \| \| --- \| |
| --- | --- |
| \| In the last three years, I falsified data in my research. \| \| --- \| |
| \| *Clarification: Falsification refers to manipulating research materials, equipment, or processes, or changing or omitting data or results such that the research is not accurately represented in the research record.*  *1. Click on the 'start' button*  *2. The circle and triangle will start alternate*  *3. Click on the 'stop' button when you are ready to answer*  *4. Choose the symbol that represents your answer* \| \| --- \| |
|  |
| \| 1 \| Circle \| \| --- \| --- \| \| 2 \| Triangle \| |

| \| End \| \| --- \| |
| --- | --- |
| \| Thank you for your participation in this survey! We are very grateful for the time you spent providing your views. We expect to be able to share first results of this study by the end of Q2 2021. All results will be available on our website: **www.nsri2020.nl**  To protect your identity optimally we had no open questions in the survey. However, if you wish to raise an issue not covered in this study, you may do so by sending us an e-mail at **info@nsri2020.nl** \| \| --- \| |
