# Supplementary material for: Prevalence of questionable research practices, research misconduct and their potential explanatory factors: A survey among academic researchers in The Netherlands
Source: PLoS One. 2022 Feb 16;17(2):e0263023. doi: 10.1371/journal.pone.0263023 (PMC8849616; doi:10.1371/journal.pone.0263023)
Supplement: Attachment — Submitted filename: PLOS One Reviewer Comments_20211221.docx [file pone.0263023.s011.docx]

PLOS One Reviewer Comments to be addressed

21 Dec 2021

Authors’ response to Reviewer Comments

Dear Editor,

Please find under each Reviewer comments, our responses as well the corresponding line numbers of the modified text. These refer to the revised manuscript *with track changes.*

We sincerely hope to hear a positive outcome of our manuscript shortly.

Best wishes

Gowri Gopalakrishna, PhD

Corresponding author

Reviewer #1: As I commented in the first review, I find this article very attractive and it has a lot of potential to be published. In this case, besides some minor changes, I think that one of the suggestions made in the first review should be reconsidered.

(1) The authors consider that they have added the psychometric properties since they have added in the supplementary materials (as also suggested by another reviewer) the correlation of the items with the total. I believe that this is insufficient considering the large sample size. I suggest that the authors make a final analysis by performing a factor analysis (if possible confirmatory) in order to justify the use of scale scores.

(2) In addition, along the same lines, the decision to combine the two scales (Mentoring and Organizational Justice scales) should also be justified with factor analysis techniques. Despite this comment, I believe that the text added by the authors helps to understand what happened. We cannot make a decision about the dimensionality of the scale just based on the value of the correlation.

***We wish to thank the reviewer for making this excellent point on the dimensionality of the scales. Accordingly, we ran a confirmatory one-factor model in Lavaan using the Weighted Least Squares Mean and Variance (WLSMV) estimator. We report these results for the explanatory factor scales in S5 Table Supplementary information.***

***As can be seen, the one-factor analysis showed excellent fit for all but one of the scales, with only the fit of the Competitiveness scale highlighting some misfit (which on inspection was caused by three items clustering together). As this is an unexpected finding, and not previously part of our preregistered data analysis plan, we therefore will consider doing explorative analysis with this finding at a later stage.***

***We also ran two and one factor models for the mentoring and organizational justice scales that we previously merged to get a sense of the dimensionality, as rightly suggested by the reviewer. In the analyses of the two original mentoring scales, we found the two factor model to fit appreciably better (CFI: 0.99, RMSEA: 0.062, SRMR: 0.051, correlation between factors>.70) than the one-factor model (CFI: 0.97, RMSEA: 0.105, SRMR: 0.086). Hence, we decided to re-run all analyses in the main manuscript that treated Survival Mentoring and Responsible Mentoring as separate explanatory factor scales.***

***As can be seen in the results of the linear prediction of QRP mean in Table 3b, this led to more interpretable results in which -as expected and inline with pertaining literature- Survival Mentoring was positively related to QRP mean, while Responsible Mentoring was negatively (albeit non-significantly) related to mean QRP use. We also fitted two and one factor models to the two original organizational justice scales (results of the separate factor analyses in S5 Table already highlighted that each 6-item scale separately fitted a one-factor model). However, in the full model with all 12 organizational justice items, the two factor model that separated the distributional justice and procedural justice (CFI: 0.99, RMSEA: 0.046, SRMR: 0.043, correlation between factors: >.98) fitted just as well as the one-factor model (CFI: 0.99, RMSEA: 0.046, SRMR: 0.043). In light of the very high correlation between the two factors and to avoid multi-collinearity, we decided to focus on the joint 12-item organizational justice scale in the main analyses (as we did in the original submission).***

***In accordance to these results based on the analyses above, we have adjusted the following parts of the manuscript:***

- ***Text in the Methods section, lines 188-191 where we removed reference to merging the mentoring scale, and in the Discussion lines 352-60 which read as follows respectively,***

***Methods (lines 188-91): “The subscales distributional and procedural organizational justice were highly correlated (correlation factor of >0.8 [S4 Table]).”***

***Discussion (lines 352-60): “Unsurprisingly, we find a moderate yet statistically significant association between survival mentoring and higher overall QRP mean suggesting that survival mentoring may be associated with higher QRPs while an association in the opposite direction, again moderate but significant, is observed for responsible mentoring and overall QRP mean. Both results agree with an earlier study [13] which explored five different types of mentoring (including responsible and survival mentoring). Our study and that of Anderson et al [13] suggests that mentors can influence behaviour in ways that both increase (in the case of survival mentoring) or decrease (in the case of responsible mentoring) the likelihood of problematic research behaviour such as QRPs***

- ***Tables 1, 3a and 3b (Main manuscript) have also been modified based on the two mentoring subscales analyzed now as two separate scales in the regression analyses.***
- ***Supplementary information: S5 Table is also now revised to include the two mentoring subscales and the item correlations and factor loadings for all scales respectively.***

Below are some other details in case you would like to review them.

(1) NSRI comprises of four components: 11 QRPs, 11 RRPs, two FFs and 12 explanatory factor 118 scales (75 questions, detailed in S7). S7 does not exist. Revise number.

***Thank you for spotting this mistake. We have changed the numbering as this should refer to S6.***

***Lines 117 -118 now read as:***

***“NSRI comprises of four components: 11 QRPs, 11 RRPs, two FFs and 12 explanatory factor scales (75 questions, detailed in S6)”***

(2) Check brackets in line 154: they do not close.

***We have now corrected this at Line 154.***

(3)It is confusing to me that there is a Figure S2, and S2a-8 are not related to those figures.

**We have now changed Figure S2 to Figure S3 so it is separate in numbering from S2a-2e Figures.**

(4) Figure 1: a)A space is missing between N and in equal in all rectangles. B)It would be necessary that the lines start touching the rectangle and not floating.

***Thank you. These changes have been made to the affected Figure 1.***

(5) Table 1. When result is 0, please indicate 0.00

***These changes have been made to Table 1.***

(6) Table 3: I do not understand the symbols in the title as I cannot find it inside the table. If it is already in the foot, it is not necessary to add it in the title.

***Thank you for correctly pointing this out. All symbols are now only indicated in the table and their explanations as footnotes.***

Reviewer #2: Thank you for considering my suggestions and incorporating them in the revised manuscript (PONE-D-21-28638-_R1). The authors have adequately addressed all my comments and provided sufficient information. There are only a few minor clarifying/formatting comments.

I wish you much success on your future work. Thank you.

Page 6 line 121: This sentence references S7, but I am not sure it is included in this file or if it is only included on the Open Science Framework. I might have missed this, but I just want to double-check.

***Thank you for this comment. S7 should have been S6. This was a numbering mistake on our part which we have now corrected in the manuscript lines 117-118.***

Page 7 line 153: States “Cronbach alpha,’ instead this should read “Cronbach’s alpha.”

***This change has been made to line 151 of the track change manuscript.***

Page 7 line 154: If using APA 7th edition, APA suggests using brackets instead of double parentheses: “(i.e., positive correlations with publication pressure and competitiveness [S4 Table]).”

***Thank you. We have removed the double parentheses and replaced with brackets as suggested above.***

Page 9 line 197: Similar to above: “(correlation factor of > 0.8 [S4 Table]).”

***Thank you. We have removed the double parentheses and replaced with brackets as suggested above.***

Reviewer #3: Dear Authors,

I appreciate your responsiveness to the feedback. Thank you for addressing the comments.

________________________________________
